# Trends in incidence of childhood cancer in Australia, 1983–2006

**DOI:** 10.1038/sj.bjc.6605503

**Published:** 2010-01-05

**Authors:** P D Baade, D R Youlden, P C Valery, T Hassall, L Ward, A C Green, J F Aitken

**Affiliations:** 1 Viertel Centre for Research in Cancer Control, Cancer Council Queensland, 553 Gregory Terrace, Fortitude Valley, Spring Hill, QLD 4006, Australia; 2 School of Public Health, Queensland University of Technology, Victoria Park Road, Kelvin Grove, QLD 4059, Australia; 3 Queensland Institute of Medical Research, 300 Herston Road, Herston, QLD 4006, Australia; 4 The Australian Centre for International and Tropical Health, University of Queensland, Herston Road, Herston, QLD 4006, Australia; 5 Royal Children's Hospital, Herston Road, Herston, QLD 4006, Australia; 6 School of Population Health, University of Queensland, Herston Road, Herston, QLD 4006, Australia

**Keywords:** cancer incidence, paediatric, childhood, trends, leukaemia, lymphoma

## Abstract

**Background:**

There are few population-based childhood cancer registries in the world containing stage and treatment data.

**Methods:**

Data from the population-based Australian Paediatric Cancer Registry were used to calculate incidence rates during the most recent 10-year period (1997–2006) and trends in incidence between 1983 and 2006 for the 12 major diagnostic groups of the International Classification of Childhood Cancer.

**Results:**

In the period 1997–2006, there were 6184 childhood cancer (at 0–14 years) cases in Australia (157 cases per million children). The commonest cancers were leukaemia (34%), that of the central nervous system (23%) and lymphomas (10%), with incidence the highest at 0–4 years (223 cases per million). Trend analyses showed that incidence among boys for all cancers combined increased by 1.6% per year from 1983 to 1994 but have remained stable since. Incidence rates for girls consistently increased by 0.9% per year. Since 1983, there have been significant increases among boys and girls for leukaemia, and hepatic and germ-cell tumours, whereas for boys, incidence of neuroblastomas and malignant epithelial tumours has recently decreased. For all cancers and for both sexes combined, there was a consistent increase (+0.7% per year, 1983–2006) at age 0–4 years, a slight non-significant increase at 5–9 years, and at 10–14 years, an initial increase (2.7% per year, 1983–1996) followed by a slight non-significant decrease.

**Conclusion:**

Although there is some evidence of a recent plateau in cancer incidence rates in Australia for boys and older children, interpretation is difficult without a better understanding of what underlies the changes reported.

Although childhood cancers (0–14 years) are rare, they were the second commonest cause of death (17% of deaths) among Australian children aged 1–14 years in 2004–2006, after injuries (Australian Institute of Health and Welfare, 2009).

In addition to the loss of life, the burden of childhood cancer extends to the long-term adverse health effects experienced by a large proportion of childhood cancer survivors, either because of the cancer itself or its treatment. (Hudson *et al*, 2003; Aziz *et al*, 2006; Goldsby *et al*, 2006; Maule *et al*, 2007; Kurt *et al*, 2008; Mertens *et al*, 2008; Oeffinger *et al*, 2008) There is also some evidence that childhood cancer and its treatment can have persisting negative effects on parents (Hardy *et al*, 2008) in relation to both finance and lifestyle(Cohn *et al*, 2003).

Children's cancer registries need to be specially designed (Cole, 2004). The Australian Paediatric Cancer Registry (APCR), one of the few population-based childhood cancer registries in the world, was first established in 1977, with full population-based coverage from 1983. Although information on cancer for all ages is available through State and Territory Cancer Registries within Australia, no other Australian registry collects detailed information on the stage of disease and treatment of childhood cancer. Such information is required for setting and measuring standards of care for children with cancer and to track changes in outcome over time.

Following a report on rising incidence rates for childhood cancer in Australia between 1982 and 1991 (McWhirter *et al*, 1996), this paper reports the latest Australian population-based incidence rates and long-term trends up to 2006 using the current International Classification of Childhood Cancers.

## Materials and methods

Notification of invasive cancer is a statutory requirement for all public and private hospitals and pathology services in Australia; therefore, the incidence data reported here are considered to represent the entire Australian population aged 0–14 years (approximately 4 million children in 2006). Cancer notifications are sent initially to state- and territory-based cancer registries, and then, for most states and territories, these notifications are transferred directly to the APCR. In those states in which legislation precludes this direct transfer, diagnostic information is accessed by the treating hospital in consultation with the state cancer registry to ensure complete enumeration. Confirmation and validation of cancer records is achieved through site visits by the APCR Data Manager to major children's hospitals throughout Australia, when information on clinical characteristics and treatment is extracted from patients’ charts.

In contrast to the coding system for adult cancers that is based on site, the internationally recognised childhood cancer classification is based on morphology (Steliarova-Foucher *et al*, 2005b). The current standard for childhood cancer is the International Classification of Childhood Cancers (ICCC-3) (Steliarova-Foucher *et al*, 2005b), which classifies tumours coded according to the ICD-O-3 nomenclature into the 12 major diagnostic groups shown in Table 1 (Steliarova-Foucher *et al*, 2005b).

Although tumours of benign or uncertain behaviour are generally not reported for adults, the ICCC-3 includes non-malignant intracranial and intraspinal tumours in categories III and X (see Tables 1 and 2) (Steliarova-Foucher *et al*, 2005b). In accordance with this accepted classification throughout this paper, childhood cancer refers to all malignant tumours, as well as to intracranial and intraspinal tumours of benign or uncertain behaviour. In those Australian states that do not collect information on tumours of benign or uncertain behaviour, such cases were captured through the major paediatric treating hospitals in that state.

Numerical indices of data quality were calculated for the diagnostic criteria of histological verification (HV) and death certificate only (DCO) (Bray and Parkin, 2009). Histological verification includes diagnoses based on histology of primary, exfoliative cytology and haematological examination of peripheral blood, histology of metastasis and autopsy with histology. These indices were calculated separately for two 10-year periods (1987–96 and 1997–2006).

Incidence rates were calculated for each cancer category, separately for each sex and age group (0–4 years, 5–9 years and 10–14 years) over the most recent 10-year period (1997–2006). Rates were age standardised to the WHO World Standard Population (Ahmad *et al*, 2001) and expressed per million population.

We used JoinPoint (National Cancer Institute, 2008) software to examine trends in incidence rates from 1983 to 2006, specifically to assess whether the magnitude or direction of trend changed during this period, and to quantify the annual percentage change (APC). To reduce the likelihood of reporting spurious changes in trends, we used a maximum of two joinpoints (i.e., up to three different trends), with a minimum of 8 years between joinpoints. The trend lines that provided the best fit to observed data, based on Monte Carlo permutation tests, were selected.

## Results

During the period 1983–2006, 13 925 childhood cancers were diagnosed in Australia; 95.4% of records were based on histological verification (HV), including 74.0% based on histology of primary, 20.7% on cytology or haematology, 0.3% on histology of metastasis and 0.4% on autopsy with histology. Of the remainder, most were clinical investigations (3.9% of total) or clinical only (0.2%). Less than 0.2% of diagnoses were based on death certificate only, with 0.3% having unknown histology. Between 1987 and 1996 and 1997 and 2006, there was a reduction in the percentage of records based on HV, from 96.8 to 93.9% (Table 1). This was mainly because of an increase in the proportion of records based on clinical investigations, from 2.9 to 5.1% for all childhood cancers, from 10.0 to 17.2% for central nervous system (CNS) and from 7.7 to 12.2% for retinoblastoma; the reasons are unclear.

In the most recent 10-year period, 1997–2006, a total of 6184 children under the age of 15 years were diagnosed with cancer in Australia (Table 2), equivalent to an annual age-standardised rate of 157 cases per million. Nine in 10 (91%) of these cancers were malignant. The remainder, 568 tumours, or 14 cases per million population were of benign or uncertain behaviour in the brain or central nervous system; the commonest types were leukaemia (34%), CNS (23%) and lymphomas (10%).

There was a 1.14:1 male/female ratio for overall childhood cancer incidence (Table 3), with a marked difference between age groups. The incidence rate of childhood cancer was higher among children aged 0–4 years (223 cases per million population) than at ages 5–9 (117 per million) or 10–14 years (131 per million). Some exceptions to this age-specific pattern were shown by lymphomas, malignant bone tumours and malignant epithelial tumours and melanomas in which incidence was highest at 10–14 years.

**Figure 1:**
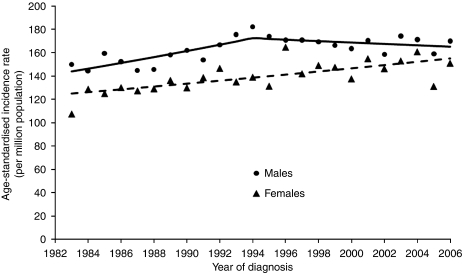
Trends in directly age-standardised (world population, per million) incidence rates for childhood cancer in Australia between 1983 and 2006. Trends modelled using Joinpoint regression.

Childhood cancer incidence increased between 1983 and 1994 for boys (+1.6% per year), but has remained stable since then (Figure 1; Table 4). The overall trend among girls increased by an average of 0.9% per year over the entire period, 1983–2006 (Table 4).

Trends varied substantially by cancer type, although the small number of specific cancers often resulted in substantial year-to-year random fluctuations in rates even when the underlying trend was statistically significant. Notably, incidence trends among both boys and girls for leukaemia, hepatic tumours and germ cell tumours showed significant increases, whereas there have been recent, significant decreases among boys in the incidence of neuroblastomas and malignant epithelial tumours and melanomas (Table 4). Owing to very small numbers (1.2 cases per year) of ‘other and unspecified malignant neoplasms’, we did not assess trends for this category.

There was also some variation in incidence rate trends by age group (Table 4). At 0–4 years, the average increase (+0.7% per year) was consistent upto 2006, but at 5–9 years there was evidence of a consistent, small, but not significant increase over time; at 10–14 years, the initial significant increase of 2.7% per year peaked in 1996, followed by a small, but not significant decrease in incidence.

## Discussion

Whereas direct comparisons with international incidence rates can be problematic because of different population standards and disease classifications, the world age-standardised incidence rates we report here (158 per million) are among the highest reported internationally (Desandes *et al*, 2004; Steliarova-Foucher *et al*, 2005a; Stack *et al*, 2007; Li *et al*, 2008; Linabery and Ross, 2008; Ocheni *et al*, 2008; Spix *et al*, 2008; Swaminathan *et al*, 2008). This is consistent with the finding that incidence shows a strong positive association with the national per capita gross income levels (Howard *et al*, 2008).

The distribution of paediatric cancer was similar to that reported in other countries, particularly in more developed countries. Leukaemia made up about a third of paediatric cancers in Australia, whereas international percentages ranged from 27% of paediatric cancers in the United States, (Linabery and Ross, 2008) 30% in Ireland (Stack *et al*, 2007) and France, (Desandes *et al*, 2004) 33% in Germany (Spix *et al*, 2008) and 35% in Shanghai, China (Bao *et al*, 2009) and Chennai, India (Swaminathan *et al*, 2008). For most countries, including Australia, the next two commonest childhood cancers were CNS (20–27%) and lymphoma (8–15%). An exception to this was in Chennai, India, where lymphomas (20%) were more common than CNS (11%) (Swaminathan *et al*, 2008).

There have been widespread reports of an increase in childhood cancer internationally since the 1970s, increasing annually by 0.6% per year from 1975 to 2002 in the United States (Ward *et al*, 2006), by 1.0% in Europe between 1970 and 1999 (Steliarova-Foucher *et al*, 2004), by 1.0% in Sweden between 1960 and 1998 (Dreifaldt *et al*, 2004) and by 0.8% per year in Western Germany (1987–2004). The much higher increase in rates in Eastern Germany (2.1% per year in 1991–2004) was probably influenced by incomplete data early in the reporting period (Spix *et al*, 2008). A subsequent report from the United States (Linabery and Ross, 2008) found evidence of a plateau in incidence trends, with a non-significant annual increase of 0.4% in rates between 1992 and 2004. Our findings in Australia are consistent with this latest report, with an increasing incidence of childhood cancer in Australia during the 1980s and mid-1990s, followed by a levelling off in the overall rate, largely because of the patterns among boys and older children.

Any discussion of the role of environmental or other risk factors in the observed trends in childhood cancer is hampered by the current limited understanding of its aetiology. Although genetic syndromes and higher birth weight are well-established risk factors, collectively they account for only a small proportion of cases (Martin *et al*, 2005; Johnson *et al*, 2009).

Changes in trends could also be due to changes in diagnostic, coding or registration practices (Howard *et al*, 2008; Spector and Linabery, 2009). For example, in the United States, the use of improved imaging technology in diagnosis has been suggested to explain the increase in childhood brain tumours during the 1980s (Smith *et al*, 1998). Our study period did not allow us to ascertain whether a similar effect held in Australia. Second, registration is often incomplete at the start of a population-based registry. A 7-year ‘run-in period’ has been suggested as ideal for such registries (Spix *et al*, 2008) and this is consistent with our data, with the establishment of the APCR in 1977, 6 years before full population-based reporting started in 1983.

It has also been suggested that the observed trends could, in part, be an artefact of reductions in infant mortality, whereby the proportion of children at greater risk of various diseases, including cancer, might be increased (Steliarova-Foucher *et al*, 2005a). In Australia, the infant mortality rate has reduced from 9.9 infant deaths/1000 live births in 1985 to 4.7/1000 in 2005. (ABS, 2007; Australian Institute of Health and Welfare, 2009), with some suggestion of a levelling off from 1998 onwards (Australian Institute of Health and Welfare, 2009). However, it is only speculative whether this has a direct association with the recent plateau for childhood cancer incidence.

As in most developed countries, average age at first birth has increased over recent decades, and there is some evidence, although inconsistent, of a positive association between maternal age at birth and risk of childhood cancer. A large US case–control study using pooled population-based data found, after adjusting for potential confounders, an 8% increase in overall childhood cancer risk for each 5-year increase in maternal age, with similar increases for most of the common subtypes (Johnson *et al*, 2009). A Swedish cohort study found a similar effect of parental age, but only for children diagnosed below 5 years of age (Yip *et al*, 2006). However, there is evidence that the strength of this maternal age effect has reduced in recent years, with earlier studies more often finding a positive association (Johnson *et al*, 2009; Maule *et al*, 2009). Exposure to an unknown confounder (for example, an environmental factor) underlying this apparent association may have changed over time.

The main strength of the APCR is its complete population coverage, notification being required by law. Notifications are sourced from a number of agencies, including cancer registries and hospital facilities, and are matched against the National Death Index held by the Australian Institute of Health and Welfare. Cases are then confirmed and validated as part of the standard APCR validation processes. There is also quantitative evidence of high data quality, based on the quantification of the DCO and HV indices. Because some of the cancers are rare, random fluctuations in rates may spuriously appear as significant trends, or alternatively it may seem that statistical power is insufficient to detect real trends. We have attempted to highlight only real trends by using conservative parameters in the JoinPoint analysis, but trends with wide confidence intervals should be interpreted with caution.

It is encouraging that there is some evidence of a plateau or reduction in childhood cancer incidence rates in Australia, driven largely by rates among boys, although we have limited understanding of what is driving these changes. The incidence changes reported here may reflect random variation or changes in unknown risk factors, and therefore highlight the need for more research into the aetiology of childhood cancer.

## Figures and Tables

**Table 1:** Indices of data quality by diagnostic group

		**1987–1996**		**1997–2006**	
**Diagnostic group**	**Cases 1983–2006**	**%DCO** a	**%HV** b	**%DCO** a	**%HV** b
All cancers	13 925	0.1	96.8	0.1	93.9
I. Leukaemias, myeloproliferative diseases, and myelodysplastic diseases (‘Leukaemias’)	4591	0.1	99.7	0.1	98.7
II. Lymphomas and reticuloendothelial neoplasms (‘Lymphomas’)	1374	0.2	99.3	0.0	99.4
III. CNS and miscellaneous intracranial and intraspinal neoplasms (‘CNS’)^c^	3158	0.3	89.5	0.0	81.4
IV. Neuroblastoma and other peripheral nervous cell tumours (‘Neuroblastoma’)	869	0.0	96.1	0.0	97.0
V. Retinoblastomas	357	0.0	91.5	0.0	82.4
VI. Renal tumours	735	0.0	100.0	0.0	97.9
VII. Hepatic tumours	174	1.6	98.4	1.0	94.9
VIII. Malignant bone tumours	602	0.0	99.6	0.0	97.3
IX. Soft tissue and other extraosseous sarcomas (‘Soft tissue’)	820	0.0	98.9	0.0	97.6
X. Germ cell tumours, trophoblastic tumours, and neoplasms of gonads (‘Germ cell’)^c^	511	0.0	97.0	0.8	93.6
XI. Other malignant epithelial neoplasms and malignant melanomas	705	0.0	99.7	0.3	98.8
XII. Other and unspecified malignant neoplasms	29	0.0	100.0	0.0	85.7

a HV = Histological vertification.

b DCO = Death certificate only.

c Category includes tumours of benign or uncertain behaviour.

**Table 2:** Incidence by diagnostic group for childhood cancer, Australia, 1997–2006^a^^,^^b^

**Diagnostic group/subgroup**	**Average number of cases per year**	**(%)**	**Rate per million population per year (95% CI)**
All cancers	618.4	100.0	157.5 (153.6–161.5)
			
*I. Leukaemias, myeloproliferative diseases, and myelodysplastic diseases (‘Leukaemias’)*	207.2	33.5	53.1 (50.8–55.4)
a. Lymphoid leukaemias	159.0	25.7	40.8 (38.8–42.8)
b. Acute myeloid leukaemias	34.3	5.5	8.7 (7.8–9.7)
c. Chronic myeloproliferative diseases	6.9	1.1	1.7 (1.4–2.2)
d. Other myeloproliferative diseases	4.8	0.8	1.2 (0.9–1.6)
e. Other and unspecified leukaemias	2.2	0.4	0.6 (0.4–0.9)
			
*II. Lymphomas and reticuloendothelial neoplasms (‘Lymphomas’)*	62.0	10.0	15.4 (14.2–16.7)
a. Hodgkin's lymphomas	26.0	4.2	6.4 (5.6–7.2)
b. Non-Hodgkin's lymphomas (excl. Burkitt lymphomas)	21.8	3.5	5.4 (4.8–6.2)
c. Burkitt lymphomas	12.4	2.0	3.1 (2.6–3.7)
d. Miscellaneous lymphoreticular neoplasms	1.1	0.2	0.3 (0.1–0.5)
e. Unspecified lymphomas	0.7	0.1	0.2 (0.1–0.4)
			
*III. CNS and miscellaneous intracranial and intraspinal neoplasms (‘CNS’)* c	140.5	22.7	35.7 (33.8–37.6)
a. Ependyomas and choroid plexus tumours^c^	13.1	2.1	3.4 (2.8–4.0)
b. Astrocytomas^c^	63.9	10.3	16.2 (15.0–17.5)
c. Intracranial and intraspinal embryonal tumours^c^	28.0	4.5	7.2 (6.3–8.0)
d. Other gliomas^c^	16.5	2.7	4.2 (3.5–4.8)
e. Other specified intracranial and intraspinal neoplasms^c^	15.5	2.5	3.9 (3.3–4.6)
f. Unspecified intracranial and intraspinal neoplasms^c^	3.5	0.6	0.9 (0.6–1.2)
			
*IV. Neuroblastoma and other peripheral nervous cell tumours (‘Neuroblastomas’)*	36.6	5.9	9.6 (8.7–10.7)
a. Neuroblastomas and ganglioneuroblastomas	36.0	5.8	9.5 (8.5–10.5)
b. Other peripheral nervous cell tumours	0.6	0.1	0.2 (0.1–0.3)
			
*V. Retinoblastomas*	14.8	2.4	3.9 (3.3–4.6)
			
*VI. Renal tumours*	32.5	5.3	8.5 (7.6–9.5)
a. Nephroblastomas and other nonepithelial renal tumours	31.2	5.0	8.2 (7.3–9.1)
b. Renal carcinomas	1.1	0.2	0.3 (0.1–0.5)
c. Unspecified renal tumours	0.2	0.0	0.1 (0.0–0.2)
*VII. Hepatic tumours*	9.8	1.6	2.6 (2.1–3.1)
a. Hepatoblastomas	8.0	1.3	2.1 (1.7–2.6)
b. Hepatic carcinomas	1.6	0.3	0.4 (0.2–0.6)
c. Unspecified hepatic tumours	0.2	0.0	0.1 (0.0–0.2)
			
*VIII. Malignant bone tumours*	26.3	4.3	6.5 (5.7–7.3)
a. Osteosarcomas	12.1	2.0	3.0 (2.5–3.5)
b. Chondrosarcomas	0.4	0.1	0.1 (0.0–0.2)
c. Ewing tumours and related bone sarcomas	12.5	2.0	3.1 (2.6–3.7)
d. Other specified bone tumours	0.7	0.1	0.2 (0.1–0.4)
e. Unspecified bone tumours	0.6	0.1	0.1 (0.1–0.3)
			
*IX. Soft tissue and other extraosseous sarcomas (‘Soft tissue’)*	33.2	5.4	8.4 (7.5–9.4)
a. Rhabdomyosarcomas	16.5	2.7	4.2 (3.6–4.9)
b. Fibrosarcomas and other fibrous neoplasms	3.1	0.5	0.8 (0.5–1.1)
c. Kaposi sarcomas	0.0	0.0	—
d. Other specified soft tissue sarcomas	11.8	1.9	2.9 (2.4–3.5)
e. Unspecified soft tissue sarcomas	1.8	0.3	0.5 (0.3–0.7)
			
*X. Germ cell tumors, trophoblastic tumours, and neoplasms of gonads (‘Germ cell’)* c	25.1	4.1	6.4 (5.7–7.3)
a. Intracranial and intraspinal germ cell tumours^c^	7.1	1.1	1.8 (1.4–2.3)
b. Extracranial and extragonodal germ cell tumours	7.7	1.2	2.0 (1.6–2.5)
c. Gonadal germ cell tumours	9.7	1.6	2.5 (2.0–3.0)
d. Gonadal carcinomas	0.5	0.1	0.1 (0.0–0.3)
e. Other and unspecified gonodal tumours	0.1	0.0	0.0 (0.0–0.1)
			
*XI. Other malignant epithelial neoplasms and malignant melanomas*	29.0	4.7	7.1 (6.3–8.0)
a. Adrenocortical carcinomas	1.3	0.2	0.3 (0.2–0.6)
b. Thyroid carcinomas	5.3	0.9	1.3 (1.0–1.7)
c. Nasopharyngeal carcinomas	1.0	0.2	0.2 (0.1–0.4)
d. Melanomas	14.3	2.3	3.5 (3.0–4.1)
e. Skin carcinomas	1.0	0.2	0.2 (0.1–0.5)
f. Other and unspecified carcinomas	6.1	1.0	1.5 (1.1–1.9)
			
*XII. Other and unspecified malignant neoplasms*	1.4	0.2	0.4 (0.2–0.6)
a. Other specified malignant tumours	1.0	0.2	0.3 (0.1–0.5)
b. Other unspecified malignant tumours	0.4	0.1	0.1 (0.0–0.3)

a Diagnostic groups defined using the International Classification of Childhood Cancers (ICCC-3) Goldsby *et al*, 2006.

b Rates age-standardised to the WHO World Standard Population Howard *et al*, 2008.

c Category includes tumours of benign or uncertain behaviour.

**Table 3:** Average annual incidence by sex and 5-year age group, Australia, 1997–2006^a^^,^^b^

**Diagnostic group**	**Males**	**Females**	**0–4 years**	**5–9 years**	**10–14 years**
*All childhood cancers*					
Average cases per year	336.7	281.7	284.6	156.4	177.4
Rate per million	167.2	147.2	222.9	117.2	130.8
95% CI	(161.6–173.0)	(141.8–152.8)	(214.8–231.2)	(111.4–123.1)	(124.8–137.0)
					
*I. Leukaemias*					
Average cases per year	112.9	94.3	108.0	55.2	44.0
Rate per million	56.4	49.6	84.6	41.4	32.4
95% CI	(53.1–59.8)	(46.5–52.9)	(79.6–89.8)	(38.0–44.9)	(29.5–35.6)
					
*II. Lymphomas*					
Average cases per year	42.4	19.6	10.2	19.1	32.7
Rate per million	20.6	10.0	8.0	14.3	24.1
95% CI	(18.7–22.6)	(8.6–11.4)	(6.5–9.7)	(12.4–16.5)	(21.6–26.9)
					
*III. CNS* c					
Average cases per year	75.4	65.1	55.8	47.6	37.1
Rate per million	37.3	33.9	43.7	35.7	27.4
95% CI	(34.7–40.0)	(31.4–36.6)	(40.2–47.5)	(32.5–39.0)	(24.6–30.3)
					
*IV. Neuroblastomas*					
Average cases per year	18.7	17.9	32.0	3.5	1.1
Rate per million	9.6	9.7	25.1	2.6	0.8
95% CI	(8.3–11.1)	(8.3–11.2)	(22.4–28.0)	(1.8–3.6)	(0.4–1.5)
					
*V. Retinoblastomas*					
Average cases per year	8.9	5.9	14.0	0.8	0.0
Rate per million	4.6	3.2	11.0	0.6	—
95% CI	(3.7–5.6)	(2.4–4.1)	(9.2–12.9)	(0.3–1.2)	—
					
*VI. Renal tumours*					
Average cases per year	14.4	18.1	24.8	5.9	1.8
Rate per million	7.3	9.7	19.4	4.4	1.3
95% CI	(6.2–8.6)	(8.3–11.2)	(17.1–22.0)	(3.4–5.7)	(0.8–2.1)
					
*VII. Hepatic tumours*					
Average cases per year	6.2	3.6	7.7	1.0	1.1
Rate per million	3.2	1.9	6.0	0.7	0.8
95% CI	(2.4–4.0)	(1.4–2.7)	(4.8–7.5)	(0.4–1.4)	(0.4–1.5)
					
*VIII. Malignant bone tumours*					
Average cases per year	13.1	13.2	2.5	6.6	17.2
Rate per million	6.3	6.7	2.0	4.9	12.7
95% CI	(5.3–7.5)	(5.6–7.9)	(1.3–2.9)	(3.8–6.3)	(10.9–14.7)
					
*IX. Soft tissue sarcomas*					
Average cases per year	18.7	14.5	12.8	8.7	11.7
Rate per million	9.3	7.5	10.0	6.5	8.6
95% CI	(8.0–10.7)	(6.3–8.8)	(8.4–11.9)	(5.2–8.0)	(7.1–10.3)
					
*X. Germ cell tumours* c					
Average cases per year	12.4	12.7	13.8	3.2	8.1
Rate per million	6.3	6.6	10.8	2.4	6.0
95% CI	(5.2–7.5)	(5.5–7.9)	(9.1–12.8)	(1.6–3.4)	6.0 (4.7–7.4)
					
*XI. Malignant epithelial tumours and melanomas*					
Average cases per year	12.6	16.4	2.3	4.8	21.9
Rate per million	6.0	8.3	1.8	3.6	16.1
95% CI	(5.0–7.2)	(7.0–9.6)	(1.1–2.7)	(2.7–4.8)	(14.1–18.4)
					
*XII. Other and unspecified malignant neoplasms*					
Average cases per year	1.0	0.4	0.7	0.0	0.7
Rate per million	0.5	0.2	0.5	—	0.5
95% CI	(0.2–0.9)	(0.1–0.5)	(0.2–1.1)	—	(0.2–1.1)

a Diagnostic groups defined using the International Classification of Childhood Cancers (ICCC-3) Goldsby *et al*, 2006.

b Rates age-standardised to the WHO World Standard Population Howard *et al*, 2008.

c Category includes tumours of benign or uncertain behaviour.

**Table 4:** Total incidence counts and annual percentage change (APC) in incidence rates of childhood cancer by diagnostic group and sex, Australia, 1983–2006^a^^,^^b^

		**Trend 1**		**Trend 2**		**Trend 3**	
**Diagnostic group**	**Total number of cases**	**Years**	**APC (95% CI)**	**Years**	**APC (95% CI)**	**Years**	**APC (95% CI)**
All children							
All cancers	13 925	**1983–1994**	**+1.7** (**+0.9,+2.5)**	1994–2006	−0.1 (−0.7,+0.6)		
0–4 years	6454	1983–2006	+0.7 (+0.3, +1.1)				
5–9 years	3525	1983–2006	+0.5 (−0.1, +1.1)				
10–14 years	3946	1983–1996	+2.7 (+1.1, +4.2)	1997–2006	−1.4 (−3.3, +0.7)		
Leukaemias	4591	**1983–2006**	**+0.9** (**+0.3,+1.5)**				
Lymphomas	1374	**1983–2006**	**+0.7** (**+0.0,+1.3)**				
CNS	3158	**1983–1998**	**+1.7** (**+0.6,+2.8)**	1998–2006	−1.8 (−4.5,+1.0)		
Neuroblastomas	869	1983–2006	+0.2 (−1.1,+1.4)				
Retinoblastomas	357	1983–2006	+0.1 (−1.1,+1.4)				
Renal tumours	735	1983–2006	+0.4 (−0.7,+1.6)				
Hepatic tumours	174	**1983–2006**	**+3.3** (**+0.8,+5.9)**				
Malignant bone tumours	602	1983–2006	+0.3 (−0.8,+1.3)				
Soft tissue sarcomas	820	1983–2006	−0.2 (−1.4,+1.1)				
Germ cell tumours	511	**1983–2006**	**+2.3** (**+0.9,+3.7)**				
Malignant epithelial tumours and melanoma	705	**1983–1996**	**+4.3** (**+1.6,+7.0)**	**1996–2006**	**−5.7** (**−9.1,−2.2)**		
							
Boys							
All cancers	7684	**1983–1994**	**+1.6** (**+0.8,+2.4)**	1994–2006	−0.4 (−1.0,+0.3)		
Leukaemias	2545	**1983–2006**	**+0.6** (**+0.1,+1.2)**				
Lymphomas	960	1983–2006	+0.5 (−0.3,+1.3)				
CNS	1685	1983–2006	+0.7 (−0.1,+1.5)				
Neuroblastomas	483	1983–1994	+3.0 (−0.8,+7.0)	**1994–2006**	**−4.1** (**−7.4,−0.6)**		
Retinoblastomas	211	1983–2006	+0.1 (−1.7,+2.0)				
Renal tumours	336	1983–2006	+0.1 (−1.6,+1.8)				
Hepatic tumours	106	**1983–2006**	**+3.7** (**+0.5,+7.0)**				
Malignant bone tumours	319	1983–2006	+0.1 (−1.3,+1.6)				
Soft tissue sarcomas	452	1983–2006	−0.5 (−2.0,+1.1)				
Germ cell tumours	253	**1983–2006**	**+2.6** (**+0.8,+4.5)**				
Malignant epithelial tumours and melanoma	318	1983–1996	+4.3 (−0.7,+9.5)	**1996–2006**	**−7.0** (**−13.0,−0.6)**		
							
Girls							
All cancers	6241	**1983–2006**	**+0.9** (**+0.5,+1.4)**				
Leukaemias	2046	**1983–2006**	**+1.3** (**+0.8,+1.9)**				
Lymphomas	414	1983–2006	+1.2 (−0.2,+2.5)				
CNS	1473	1983–2006	+0.5 (−0.3,+1.4)				
Neuroblastomas	386	1983–2006	+1.3 (−0.5,+3.1)				
Retinoblastomas	146	1983–2006	−0.5 (−2.4,+1.4)				
Renal tumours	399	1983–2006	+0.7 (−0.7,+2.1)				
Hepatic tumours	68	**1983–2006**	**+2.5** (**+0.0,+5.0)**				
Malignant bone tumours	283	1983–2006	+0.5 (−1.0,+2.0)				
Soft tissue sarcomas	368	**1983–1992**	**+7.7** (**+0.8,+15.1)**	1992–1999	−8.0 (−18.0,+3.2)	1999–2006	+5.5 (−3.0,+14.9)
Germ cell tumours	258	**1983–2006**	**+2.0** (**+0.2,+3.9)**				
Malignant epithelial tumours and melanoma	387	1983–2006	+0.5 (−1.3,+2.5)				

a Trends based on incidence rates age-adjusted to the WHO World Standard population Howard *et al*, 2008.

b APC indicates Annual Percentage Change, with 95% confidence intervals in brackets.

^c^Trends based on incidence rates age-adjusted to the WHO World Standard population Howard *et al*, 2008.

^d^APC indicates Annual Percentage Change, with 95% confidence intervals in brackets. Bold type indicates statistical significance at the 0.05 level.
